# The Biomedical Engineering Labor Market in Greece: A Survey Investigating Job Outlook, Satisfaction and Placement

**DOI:** 10.1007/s43683-022-00088-x

**Published:** 2022-11-11

**Authors:** Dimitris Glotsos, Spiros Kostopoulos, Panagiotis Liaparinos, Pantelis Asvestas, Ioannis Kalatzis

**Affiliations:** Department of Biomedical Engineering, University of West Attica, Egaleo, Athens, Greece

**Keywords:** Biomedical engineering, Labor market, Job placement

## Abstract

In this study, we have evaluated the real-world conditions, the job outlook and the job satisfaction in the Biomedical Engineering (BME) sector in Greece on the basis of the experience of about 12% of the graduates of the BME Department of the University of West Attica, Greece. An anonymous online questionnaire, implemented on the Microsoft Forms platform using multiple choice questions, short text answers and Likert-based scales, became publicly available to the graduates of the BME department. About 12% of the department’s graduates responded to the survey. Results show that the time to first employment is very fast for both men and women. About 51.4% of men and 69.4% of women find their first job employment in the BME sector even before their graduation. The internship is considered important for first job placement by more than 50.6% of participants. BME jobs are perceived as most interesting (73.6%), in a good environment (71.9%), with satisfactory career prospects (45.9%), with satisfactory monthly net salary (44.2%) and satisfactory working hours (52.8%). Men are mostly employed in Service (40.5%), whereas women are mostly employed in Sales (33.3%). Most graduates with BSc degree are employed in Service (39.1%) and Sales (21.8%), most graduates with MSc degree are employed in Service (34.6%) and Hospitals/Health care centers (21.2%), and most graduates with PhD degree are employed in Academia and R&D (62.5%). Most well-paid participants (>1500 euros net salary) were PhD holders (71.5%), followed by MSc holders (25%) and BSc holders (16.2%). Maximum monthly salaries were found for those with more than 10 years of experience. In terms of BME sector, most well-paid participants (>1500 euros monthly net salary) are those working with R&D (86.7%), Sales (86.7%) and Management (60%). There is a high demand for biomedical engineers in the labor market in Greece, despite the continuing economic recession that the country is suffering from the past 12 years.

## Introduction

The Biomedical Engineering (BME) sector is among the biggest industrial sectors worldwide, with tens of thousands of manufacturers producing more than 500,000 different types of biomedical products.^9^ In particular, in 2020 the European medical technology industry employed over 730,000 people in 32,000 companies with a dynamic and increasing job outlook.^9^ Similarly in the US, the biomedical engineering sector, currently, is expected to increase up to 6% until 2030.^17^ Most databases and organizations are most likely to underestimate the total number of BME jobs due to the following reasons: First, we should take under consideration that many BME related jobs are still classified as non-BME jobs in some databases, i.e. bio-electronics, is considered as a branch of electronic engineering, not as a branch of biomedical engineering, biomedical informatics is considered as a branch of informatics, not as a branch of biomedical engineering etc. Second, many BME job positions are filled by non-BME degree holders. Such jobs, in some cases, are not officially counted as BME jobs. In any case, the total number of BME jobs is probably impossible to find.

The BME sector has known continuous innovations for more than a century.^4^ Among historical contributions may be considered the introduction of X-ray imaging systems in the early 1900s, the development of surgical aid technologies, such as mechanical ventilators and angiography systems in the 1930s–1940s, the production of electron microscopes in the 1950s, the invention of 3D non interventional imaging systems, such as the X-ray computed tomography in the 1960s–1970s, the rapidly evolving Magnetic Resonance Imaging technology in the 1970s, the human genome project that was completed in the 1990s, the multimodal hybrid imaging systems, such as PER/CT presented in the late 1990s, the smart hospital and smart technologies that we are currently experiencing in the modern era. Even today, the BME sector remains highly innovative. In 2019 there were about 14,000 new patent applications in EU, comprising the second largest patent application domain after digital communications.^9^

Biomedical engineers support not only the innovations of medical technology, but also its effective and safe implementation. BME jobs may be found in various sectors, such as in a/service, maintenance and repair of BME equipment, b/research, design and development of new technologies and instruments, c/sales, marketing and promotion of BME products, d/application specialist aiders, who stand next to the doctors for supporting the implementation of complex BME equipment and protocols, i.e. installation of implants in cardiovascular surgery, e/management and development of spin-off and start-ups, f/hospitals and healthcare centers, and g/academia and education.

Considering that a/modern medicine and biology rely (and evolve) on technology,^3,7,8,15^ and b/ageing of population will boost the necessity for technologies able to support the prolongation of life with good quality of living,^12^ biomedical engineers are expected to play a crucial role in the forthcoming technological era.

The COVID-19 pandemic has exploded the requirements for biomedical engineers in the labor market, since management of the pandemic profoundly relies on biomedical technologies. For example, treatment of patients with serious complications may require hospitalization at an Intensive Care Unit, which relies on technologies, such as the vital signs monitors, the mechanical ventilators, the gas/oxygen flow installations, the drug delivery automation, the extracorporeal blood purification, etc. Diagnosis of affected patients requires specific diagnostic examinations, which are possible due to technologies, such as the PCR instruments, the Serology/Antibody instruments, the Rapid Testing Devices, the X-ray Computed Tomography, etc. Monitoring of the virus mutations/variants, such as the Delta and the Omicron variants, requires sequencers and bioanalyzers, optical instruments and microscopy, bioinformatics, etc. Epidemiology of the pandemic requires sophisticated data analysis algorithms based on artificial intelligence and deep learning. Research on the SARS-CoV-2 requires specialized instruments and methods for the design/discovery of new vaccines and drugs. Biomedical engineers have been supporting and sustaining all of the above technologies over the last 2.5 years of the pandemic.

BME study programs are increasing rapidly worldwide to meet the high demands of the labor market and the industry for biomedical engineers able to handle, understand and keep up with the non-stop evolving biomedical technologies. Although 30–40 years ago BME was seen as a specialized scientific domain^16^ suitable for postgraduate studies, nowadays most countries have undergraduate programs in BME with modern study programs.^6,13,16,18^ The main supplier of biomedical engineers in the Greek labor market is the Department of Biomedical Engineering of the University of West Attica (UniWA), the only University Department in Greece that organizes undergraduate studies in BME from 1985.

In this study we have evaluated the real-world conditions, the job outlook and the job satisfaction in the BME sector in Greece on the basis of the experience of about 12% of the graduates of the BME Department of the UniWA. In this way, interested audience (such as students selecting college/university study programs, graduates who are looking for a career change, etc) may be informed about the real-world conditions, the employment opportunities, the employment quality and the overall career prospects in the BME sector. Moreover, this study may assist the labor market to identify strengths and pitfalls enabling fine tuning interventions to attract high quality graduates. Finally, this study may contribute towards identifying the strengths and the weaknesses of the BME Department of UniWA (or any other similar BME study program) in terms of job outlook prospects of its graduates. To the best of the authors knowledge, such an attempt for the BME sector seems to be for the first time presented in literature, with the exception of a similar study exploring BME outlook aspects in Finland in 1999.^11^

The BME Department was established in 1985 as the Department of Medical Instruments Technology of the Technological Educational Institute of Athens, Greece. In 2018 the Department was integrated with its current name at the newly founded University of West Attica, Greece, the third biggest University in Greece in terms of student enrollment with 55,700 undergraduate and 5960 postgraduate students (MSc + PhD). UniWA comprises 6 Schools (Engineering, Health & Care Sciences, Public Health, Food Sciences, Administrative, Economic and Social Sciences, Applied Arts and Culture) and 27 Departments.

## Materials and Methods

An anonymous online questionnaire was designed to quantify real-world employment conditions, satisfaction and prospects. The questionnaire was implemented on the Microsoft forms platform in Greek using multiple choice questions, short text answers and Likert scales. In particular, we have utilized two different Likert scales. The first one includes the options ‘strongly disagree’, ‘disagree’, ‘neutral’, ‘agree’, ‘strongly agree’. The second one includes the options ‘strongly dissatisfied, ‘dissatisfied, ‘neutral’, ‘satisfied, ‘strongly satisfied’. For the presentation of our findings, we have grouped a/‘strongly disagree’ with ‘disagree’, b/‘strongly agree’ with ‘agree’, c/‘strongly dissatisfied’ with ‘dissatisfied’, and d/‘strongly satisfied’ with ‘satisfied’. The results are illustrated in diverging stacked bar charts for facilitating separation between ‘positive’ and ‘negative’ opinions. The baseline of the diverging stacked bar charts are the neutral opinions (‘neither agree or disagree’, ‘neither satisfied or dissatisfied’), the negative opinions are grouped/placed to the left side of the baseline (also presented with negative numbers) and the positive opinions are grouped/placed to the right side of the baseline right the positive opinions (also presented with positive numbers).

The questionnaire included five main sections:

*Demographics:* basic demographic data were collected, such as age, sex and city of residence.

*Educational profile:* the educational profile was documented (graduate, postgraduate, MSc, PhD).

*Working profile:* the working profile was requested in terms of years of experience, placement in different BME sectors (i.e. service, sales, hospitals, research etc), employment activities (i.e. software, hardware) and number of different BME placements till today.

*Job satisfaction:* the job satisfaction was assessed in terms of monthly salary, working hours, working environment, job interest and career prospects.

*BME studies and job placement:* this section investigates how easy is to find job placement for those holding BME degrees.

The questionnaire became publicly available to the graduates of the BME Department of UniWA after receiving approval by the Research Ethics Committee of the UniWA (Approval number 4132-26/01/2022). On average, the Department has 60 graduates/year. The first students graduated in 1989, 4 years after the establishment of the department in 1985. According to the latest available data before the pandemic in 2018, graduates per year have increased to 71.

The survey was communicated by a/Email announcements, b/Facebook announcements, c/LinkedIn announcements, and d/Websites announcements.

The survey was completed in February 2022 collecting in total 233 responses. Two hundred twenty-seven (227) participants were graduates of the BME Department of UniWA (12% of the total number of graduates of the Department from 1985 to 2021). The remaining six (6) responses were graduates of other universities, among which, two (2) responses from universities outside Greece. Due to the limited number of the latter group, only the 227 responses from the BME Department of UniWA were retained for further analysis. From the 227 responses, only 4 participants declared that they never had employment in the biomedical sector. The remaining 223 responses have had, till February 2022, at least one job placement in the biomedical sector.

## Results

### Demographics

The participants are 76.0% men and 24.0% women. This statistic is, more or less, the same to the distribution of male and female BME graduates in Greece for the period 1985–2002 (76–82% males vs 18–24% females). The demographics of the survey responders and the demographics of BME students of the Biomedical Engineering Department are more or less the same in terms of gender (76% men between survey responders, 79% men between BME graduates). The age groups were 6.0% < 25 years, 71.0% 25–40 years and 23.0% 41–55 years. Most of the participants were employed in Athens (84.0%).

### Participant’s Educational Profile

The education profile of participants is 59.0% BSc graduates, 35.0% MSc graduates and 6.0% PhD graduates.

### Participant’s Working Profile

Participants’ working experience ranges from starters (< 3 years, 21.7%), early stage (3–5 years, 28.6%), medium experienced (6–10 years, 19.0%), experienced (11–20 years, 23.1%) to very experienced (> 21 years, 7.5%) (Table 1). The placement of the participants to different BME sectors is 37.0% in Service, 19.4% in Sales, 12.3% as Applications specialists, 11.0% in Hospitals/Private clinics/Health centers, 8.4% in other, unclassified positions related to BME, 5.7% in Management, and 6.2% in Research and Academia (Table 2). The employment in different BME sectors by gender and study level shows that men are mostly employed in Service (40.5%), whereas women are mostly employed in Sales (33.3%). Most graduates with BSc degree are employed in Service (39.1%) and Sales (21.8%), most graduates with MSc degree are employed in Service (34.6%) and Hospitals/Health care centers (21.2%), and most graduates with PhD degrees are employed in Academia, Research and Development (62.5%).

**Table 1:** Years of employment experience in the BME sector by gender and study level.

Years of experience	Men (%)	Women (%)	BSc (%)	MSc (%)	PhD (%)
< 1 year	10.5	5.1	12.6	16.9	0
1–2 years	15.3	6.1	16.1	24.7	0
3–5 years	25.8	42.9	31	18.2	12.5
6–10 years	18.5	28.6	14.9	18.2	12.5
11–20 years	21	8.2	23	14.3	37.5
> 21 years	8.9	9.2	2.3	7.8	37.5

**Table 2:** Placement in different BME sectors by gender and study level.

BME sectors	Men (%)	Women (%)	BSc (%)	MSc (%)	PhD (%)
Research and development	3.6	0	0	5.8	12.5
Academia	3.6	2.8	0	1.9	50
Management	6.3	13.9	9.2	3.8	25
Other	6.3	8.3	9.2	3.8	0
Hospital/clinic/Health center	11.7	16.7	8	21.2	12.5
Application specialist	12.6	5.6	12.6	9.6	0
Sales	15.3	33.3	21.8	19.2	0
Service	40.5	19.4	39.1	34.6	0

**Figure 1:**
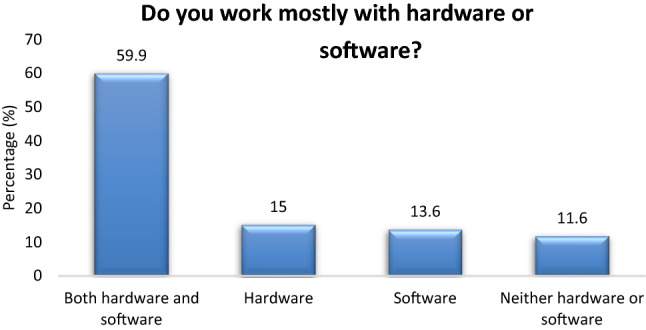
Hardware and software.

**Figure 2:**
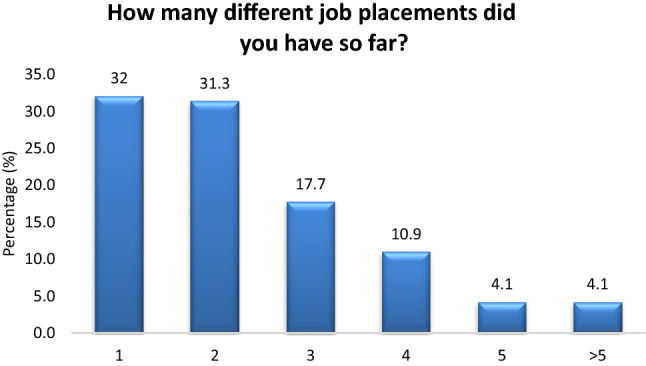
Number of different BME job placements till today.

**Figure 3:**
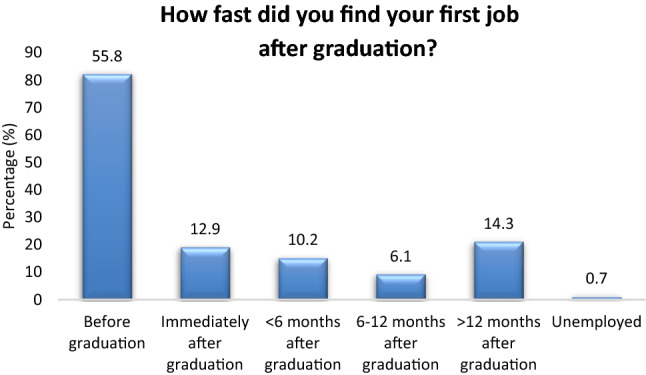
Working activities involving hardware and/or software.

Most of the participants are involved in both hardware and software (59.9%), followed by mostly hardware (15%), mostly software (13.6%) and other (11.6%) (Fig. 1). Most of the participants (63.3%) have worked in 1 or 2 different BME employers in the past (Fig. 2). Most of the participants (55.8%) find their first job placement before they graduate from the BME study program (Fig. 3). The time to first employment appears very fast for both men and women. About 51.4% of men and 69.4% of women find their first job employment before graduation. Based on study level, time to first job placement appears fastest for BSc holders (56% found placement before graduation), followed by MSc holders (53.8%) and PhD holders (37.5%). Finally, first job placement before graduation is faster for those employed in Service, Sales and as Application Specialists (63.5%, 55.2% and 50% respectively) (Table 3).

**Table 3:** Time to first job placement by gender, study level and BME sector.

Time to first job placement	Men (%)	Women (%)	BSc (%)	MSc (%)	PhD (%)	Service (%)	Sales (%)	Hospital (%)	R&D (%)	Management (%)	Application specialist
Before graduation	51.4	69.4	56	53.8	37.5	63.5	55.2	42.1	33.3	37.5	50
Immediately after graduation	14.4	8.3	14.3	9.6	12.5	11.5	17.2	10.5	11.1	37.5	6.3
< 6 months after graduation	11.7	5.6	8.8	9.6	25	15.4	3.4	0	22.2	12.5	12.5
6–12 months after graduation	6.3	5.6	9.9	7.7	0	3.8	10.3	10.5	0	0	12.5
> 12 months after graduation	15.3	11.1	9.9	19.2	25	5.8	13.8	36.8	33.3	12.5	18.8
Currently unemployed	0.9	0	1.1	0	0	–	–	–	–	–	–

### Job Satisfaction

**Figure 4:**
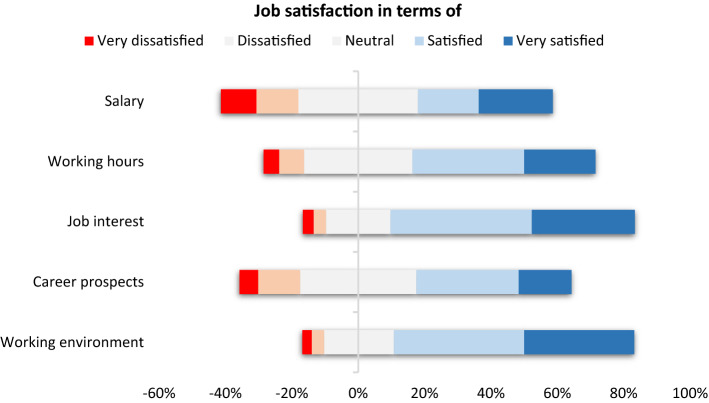
Job satisfaction in terms of monthly salary, working hours, job interest, career prospects and working environment.

In general participants declare mostly satisfied in terms of monthly salary (44.2%), working hours (52.8%), job interest (73.6%), career prospects (45.9%) and working environment (71.9%) (Fig. 4). According to Table 4, monthly salary satisfaction increases with study level (BSc 47.1%, MSc 56.4%, PhD 62.5%), whereas it remains similar for both genders (51.4%). Job interest is high for all study levels and for both genders (from 71.4 to 82.4% respectively). In terms of career prospects, BSc holders declare most satisfied (52.3%).

**Table 4:** Job satisfaction by gender and study level.

Job interest	Men (%)	Women (%)	BSc (%)	MSc (%)	PhD (%)
Satisfied or very satisfied	80.2	71.4	75.9	82.4	75
Neither satisfied or dissatisfied	13.5	22.9	18.4	11.8	12.5
Dissatisfied or very dissatisfied	6.3	5.7	5.7	5.9	12.5

Monthly salary	Men (%)	Women (%)	BSc (%)	MSc (%)	PhD (%)
Satisfied or very satisfied	51.4	51.4	47.1	56.9	62.5
Neither satisfied or dissatisfied	30.6	40	35.6	31.4	12.5
Dissatisfied or very dissatisfied	18	8.6	17.2	11.8	25

Career prospects	Men (%)	Women (%)	BSc (%)	MSc (%)	PhD (%)
Satisfied or very satisfied	49.5	45.7	52.3	44	37.5
Neither satisfied or dissatisfied	33.9	37.1	33.7	38	25
Dissatisfied or very dissatisfied	16.5	17.1	14	18	37.5

**Figure 5:**
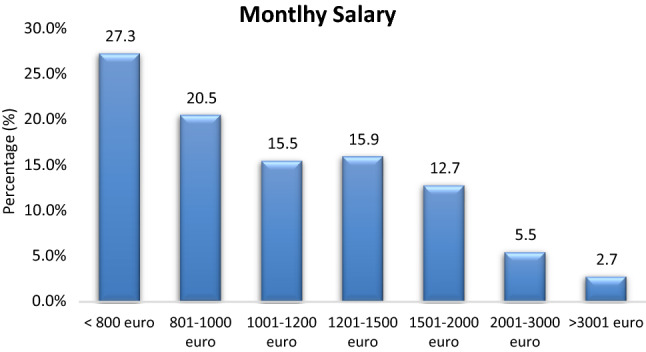
Monthly salary.

Monthly net salary shows a range from < 800 to > 2000 Euros, with the highest frequencies at the lower end (Fig. 5). About 38.5% of men have monthly net salary up to 1000 euros, whereas the respective percentage for women is 55.9%. In terms of study level, most well-paid participants (> 1500 euros monthly net salary) are PhD holders (71.5%), followed by MSc holders (25%) and BSc holders (16.2%) (Table 5). Monthly salary increases with years of experience. Maximum salaries are found for those with more than 10 years of experience (Table 6). In terms of BME sector, most well-paid (> 1500 euros net salary) are those working with R&D (86.7%), Sales (86.7%) and Management (60%) (Table 7).

**Table 5:** Salary by gender and study level.

Salary	Men (%)	Women (%)	BSc (%)	MSc (%)	PhD (%)
< 800 €	17.4	26.5	25.6	12.5	0
801–1000 €	21.1	29.4	23.3	25	14.3
1001–1200 €	16.5	20.6	16.3	18.8	0
1201–1500 €	20.2	11.8	18.6	18.8	14.3
1501–2000 €	13.8	8.8	11.6	10.4	42.9
2001–3000 €	6.4	0	2.3	10.4	0
> 3001 €	4.6	2.9	2.3	4.2	28.6

**Table 6:** Salary by years of experience.

Years of experience	< 800 € (%)	801–1000 € (%)	1001–1200 € (%)	1201–1500 € (%)	1501–2000 € (%)	2001–3000 € (%)	> 3001 € (%)
< 1 year	35.5	2.8	3.3	0	0	0	0
1–2 years	35.5	19.4	3.3	0	0	0	0
3–5 years	16.1	47.2	26.7	39.1	8.3	33.3	0
6–10 years	3.2	16.7	33.3	26.1	16.7	16.7	40
11–20 years	9.7	13.9	26.7	34.8	75	50	60
> 20 years	0	0	6.7	0	0	0	0

**Table 7:** Salary by BME sector.

Years of experience	< 800 € (%)	801–1000 € (%)	1001–1200 € (%)	1201–1500 € (%)	1501–2000 € (%)	2001–3000 € (%)	> 3001 € (%)
Service	44.8	31.3	35.5	52	50	0	0
Sales	17.2	28.1	22.6	8	20	50	16.7
Hospitals	13.8	18.8	9.7	16	0	0	0
R&D	6.9	0	0	0	20	16.7	50
Management	6.9	6.3	3.2	4	10	16.7	33.3
Application specialist	3.4	9.4	19.4	16	0	0	0
Other	6.9	6.3	9.7	4	0	16.7	0

### BME Studies and Employment

**Figure 6:**
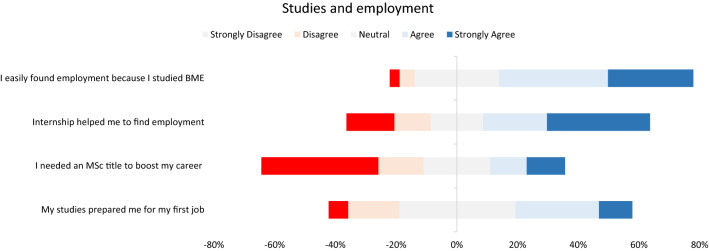
Success of studies in facilitating job placement.

Participants believe that their BME studies are most effective in assisting them to find a placement in the labor market (61.4% agree or strongly agree, 26.8% do not agree or disagree (neutral) and 9.5% disagree or strongly disagree). The internship is considered important for first job placement (50.6% agree or strongly agree, 16% do not agree or disagree (neutral) and 26% disagree or strongly disagree) (Fig. 6). The MSc degree did not appear to boost career prospects for most participants (19.9% agree or strongly agree, 16.9% do not agree or disagree (neutral) and 42% disagree or strongly disagree). The studies curriculum prepares satisfactory students for the requirements of their first job placement (38.1% agree or strongly agree, 37.2% do not agree or disagree (neutral) and 23% disagree or strongly disagree) (Fig. 6).

## Discussion

BME is one of the most innovative industrial sectors in Europe being the second biggest patent application domain in EU for 2019.^9^ BME played a crucial role during the COVID-19 pandemic. Without the support of technology, the COVID-19 pandemic would have claimed unimaginable numbers of lives.^19^ With the aid of technology,^1,2,5,14^ societies and organized health care systems managed to prevent hundreds of thousands of deaths that would have been otherwise inevitable. Biomedical technology can be found everywhere, from daily life (i.e. face masks, antigen testing, digital thermometers, oximeters, glucose meters etc), to sophisticated organized health care systems (i.e. Intensive Care Units, Radiology Departments, Artificial Kidney facilities etc). The research, design, production, maintenance, quality control, installation and implementation of such technologies in every day practice are within the numerous responsibilities of the modern biomedical engineer.

In this study we have evaluated the real-world conditions and the labor market of biomedical engineering in Greece on the basis of the experience of about 12% of the graduates of the BME Department of UniWA, the only University Department in Greece that organizes undergraduate studies in BME from 1985.

The study shows that BME graduates can easily find job placement even before their graduation. In fact, it is quite impressive that more than half of the participants (55.8%) have found their first job placement in the BME market before finishing their BME studies. This is an indication of the high demand of the market for biomedical engineers in Greece, despite the continuing economic recession that the country is suffering from the past 12 years. Most of the graduates found their first job employment in Service, Sales and as Application Specialists (Table 3).

The BME job placement is perceived as most interesting (73.6%), in a good environment (71.0%), with satisfactory career prospects (45.9%), with satisfactory net salary (44.2%) and satisfactory working hours (52.8%). Salary increased with years of experienced (maximum for > 11 years of experience) (Table 6) and study level (maximum for PhD holders) (Table 4). Higher paid positions (> 1500 euros) were found in Sales (20.0%), R&D (19.0%), Administration/management (19.0%), Service (16.0%), and for Application specialists (13.0%). Most participants in the higher paid category are of medium age (55.0% are 25–40 years). The median salary of the participants is 1001–1200 euros, whereas the minimum net salary in Greece is 586 euros.^10^

One interesting finding is that the majority of women have net salaries < 1000 euros (55.9%).On the other hand, the majority of men have net salaries > 1000 euros (61.5%). Although most women work with Sales, which we have identified as one of the highest paid BME domains, women seem to have smaller monthly salaries than men. This finding may be attributed to the experience of our participants. Very experienced participants (> 10 years of employment), which are also the most well-paid participants, were significantly lower for women than men (17.4% vs 29.9% respectively).

The role of internships is important for everyone involved, the Department of BME study program, the undergraduate students and the industry. First, internships provide technical know-how to the students. Second, students are exposed to the real-world requirements of employment in the BME sector. Third, students get to know the various job positions in the BME sector (i.e. service, sales, application specialist etc). In this way, they may identify the jobs positions that are most suitable for them. Forth, internships provide the bridge between academia and industry. Universities learn the demands of the labor market and adjust their activities, when possible, to better prepare students for the real-life employment conditions. Moreover, industry collaborates with the universities to implement educational and research programs of common interests. Fifth, internships give the appropriate time to both employers and students to decide whether their cooperation may continue even after the completion of the internship period. The latter was verified by the results of this study, which showed that almost 50% of the students have retained or upgraded their position at the same employer even after the completion of their internship period.

Participants who hold an MSc title, consider their MSc degree is a key factor for improving of their career prospects. This finding was observed for those working in Research and Development, Hospitals and Management. Indeed, for someone to follow a research career, the MSc title is a minimum requirement. For hospital engineers, the MSc title is translated into higher salaries and promotions. Finally, for those working with management, usually they have some kind of MBA MSc, which is considered as a minimum requirement for this particular field. Service engineers, engineers in Sales and Application specialists reported less importance of MSc titles in their career prospects.

Most of the biomedical engineers work with both hardware and software (59.9%). This is not surprising. The last 30 years the widespread emergence of digital biomedical systems has radically changed the demands for biomedical engineers that can handle both the hardware and the software components of the equipment. The era that the biomedical engineer was thought as an electrical and electronic engineer who is exclusively involved with electronic circuits, belongs to the distant past. Nowadays, the modern biomedical engineer should by equally familiar with both the hardware and the software components of biomedical instruments.

The BME study curriculum was considered as adequate in preparing students for their first job placement according to 35.5% of participants (22.8% considered the study curriculum as inadequate). This is more or less expected since the goal of any BME study program is to elaborate on the science, not to train students according to the requirements of the labor market. However, this doesn’t change the fact that many graduates have the opinion that their studies curriculum could have been designed more efficiently to equip them with basic skills for smooth transition to the labor market. Suggestions to improve this issue might consider a/continuous update of the study program to meet the requirements of the continuing evolving biomedical sector, b/development of targeted initiatives between the BME study programs and the labor market, c/design of specialized study tracks into the BME study programs for in depth preparation of students in a particular BME sector (i.e. in vitro, in vivo, bioelectronics, management, imaging, artificial intelligence etc), and d/strengthening of the bonds between the BME study programs and the graduates alumni to keep up to date with the evolution of the labor market.

To bring the studies closer to the labor market, the Department of Biomedical Engineering of the UniWA has organized several actions. First, the Department has organized and implemented the study presented in this paper, to better understand the employment opportunities and the real-world conditions of the BME labor market in Greece. Second, the Department has established an active collaboration with the Union of Biomedical Engineers in Greece, co-organizing seminars and workshops. Third, the Department has launched a new initiative during the last 5 years for receiving announcements of new BME Job listings from the labor market. These job listings, are, then, forwarded to the students and the alumni of the Department. Forth, the Department is currently interviewing active BME engineers that publicly share their experience with the Department’s students. Fifth, the Department has recently launched a new English taught MSc program entitled ‘Biomedical Engineering and Technology’, that aims to assist graduates with relevant background, to change their career towards the BME sector. In the aforementioned MSc program, an important aspect of educational activities will be co-organized with the participation of active biomedical engineers currently employed in the BME labor market.

The only similar study found exploring the employment outlook of BME graduates, was implemented in Finland in 1999.^11^ According to this study, the vast majority of participants (90%) found their first job placement within three months after their graduation. However, not every job placement was in BME sector. Only 50% of the above-mentioned job placements were in the BME sector. This could be explained by the fact that during the 1990s, BME was not seen as an independent scientific domain by the labor market. BME related jobs were covered mainly by electrical and electronic engineers. Today, the BME domain is identifiable by the labor market, whose first choice for BME related jobs seems to be graduates with BME degrees. Another interesting finding of that study was that job placement of graduates with MSc degree was stronger related to the BME sector than the job placement of gradates without MSc degree. Finally, the study concluded that the BME degree awarded by the Tampere University of Technology provided overall good career prospects to its graduates.

There are some limitations in this study that the reader should be aware of. One limitation is that we could not estimate potential bias from graduates that did not respond to the questionnaire because they did not have employment, or they were under-employed or they, simply, did not like their job placement in the BME sector. Another limitation is that we couldn’t collect enough responses from non-BME degree engineers working in the BME sector. It is not uncommon to find electrical and electronic engineers, software engineers, mechanical engineers and other related engineers working in the BME sector. Finally, we could not collect enough responses from participants working in the BME sector outside Greece. The above-mentioned limitations could be addressed in a future work that could involve the participation of additional BME study programs organized by universities outside Greece in order to expand our estimates on a larger geographical scale.

Studies as the one presented in this paper, are lacking from literature. We believe that such studies are important since they may assist all parties involved towards better decision making. More specifically, a/student candidates will be aware of the job placement opportunities, the overall employment quality and the career prospects of each different university/college study program, b/the university/college study programs will be able to identify their strengths and their weaknesses to enable fine-tuning modifications in terms of job outlook prospects of their graduates, c/governments and institutions responsible for designing of national educational policies, would be aware of the strengths and the weaknesses of each university/college study program in terms of job outlook, and d/the labor market would be able to identify potential improvements to attract high quality graduates. In this study we have tried to analyze the above-mentioned factors for the BME domain for the graduates of the Biomedical Engineering Department of the UniWA, Greece. The study shows that BME graduates can easily find job placement even before their graduation. The BME jobs are perceived as most interesting, in a good environment, with satisfactory career prospects, with satisfactory net salary and satisfactory working hours. There is a high demand for biomedical engineers in the labor market in Greece, despite the continuing economic recession that the country is suffering from the past 12 years.

## Data Availability

Not applicable.
